# Financial incentives for the deployment of Enhanced Recovery After Surgery (ERAS) in the SwissDRG inpatient prospective payment system: a national study

**DOI:** 10.1093/bjsopen/zraf017

**Published:** 2025-03-03

**Authors:** Gaëtan-Romain Joliat, Fabian Grass, Joachim Marti, Valérie Addor, Lucien Gardiol, Charles André Vogel, Nicolas Demartines, Fabio Agri

**Affiliations:** Department of Visceral Surgery, Lausanne University Hospital CHUV, University of Lausanne (UNIL), Lausanne, Switzerland; Department of Visceral Surgery, Lausanne University Hospital CHUV, University of Lausanne (UNIL), Lausanne, Switzerland; Center for Primary Care and Public Health (Unisanté), University of Lausanne, Lausanne, Switzerland; Department of Development and External Affairs, Lausanne University Hospital CHUV, Lausanne, Switzerland; Department of Administration and Finance, Lausanne University Hospital CHUV, Lausanne, Switzerland; Department of Administration and Finance, Lausanne University Hospital CHUV, Lausanne, Switzerland; General Direction, Lausanne University Hospital CHUV, Lausanne, Switzerland; Department of Visceral Surgery, Lausanne University Hospital CHUV, University of Lausanne (UNIL), Lausanne, Switzerland; Department of Administration and Finance, Lausanne University Hospital CHUV, Lausanne, Switzerland

Enhanced Recovery After Surgery (ERAS) protocols integrate multimodal perioperative care to improve patient recovery and outcomes^1^. Initially introduced for colorectal surgery, ERAS has since expanded across surgical specialties, with studies demonstrating reduced length of stay (LoS), fewer complications, and cost-effectiveness, particularly when used in abdominal surgery^2^.

Diagnosis-related groups (DRG) determine reimbursement in the Swiss healthcare system (SwissDRG), each DRG endorsing a resource utilization score called cost weight (CW)^3^. Under SwissDRG, short LoS outliers trigger financial deductions from standard reimbursements (inlier CW), potentially penalizing hospitals adopting ERAS due to its LoS-reducing effects^3^. This study investigated ERAS impact on LoS for patients treated at ERAS and non-ERAS hospitals in Switzerland, and financial implications from both the hospital and healthcare system perspectives.

Data were collected from a national benchmarking database of Swiss hospitals. The registry collects anonymous routine discharge-level and revenue data according to the national tariff structure, as well as costs uniformly calculated on a national scale. Stays from January to December 2022 were selected based on DRG related to pathologies of the digestive organs and hepatobiliary system/pancreas, *Supplementary materials*. LoS and cost–revenue analyses were performed. For readmissions occurring within 18 days after discharge, the associated costs were consolidated into the initial stay and therefore included. Comparisons of outcomes in academic and non-academic ERAS (ERAS-A and ERAS-NA) and non-ERAS (non-ERAS-A and non-ERAS-NA) hospitals were performed using standard statistics, *Supplementary materials*. For means calculated from variables that were already means, no standard deviation was reported.

In 2022, 248/278 (89.2%) hospitals contributed to the national benchmarking database, and 132 (53.2%) provided acute somatic care. The relevant DRG were found in 111/132 (84.1%) hospitals, of which 15 (13.5%) deployed ERAS (ERAS-A: *n* = 2 and ERAS-NA: *n* = 13), whereas 96 were non-ERAS hospitals (non-ERAS-A: *n* = 3 and non-ERAS-NA: *n* = 93). A total of 142 755 hospital stays were collected, *Supplementary materials*. Overall, ERAS hospitals had a higher low LoS outlier rate than non-ERAS hospitals (8.5% *versus* 7.1%, *P* < 0.001), and mean coverage rates were 93.5% for ERAS and 94.6% for non-ERAS (*P* < 0.001). A significantly higher mean revenue occurred in non-ERAS hospitals, but mean costs/case were significantly lower in ERAS hospitals. Comparison of hospitals of the same size and scope is summarized in *Table 1*.

**Table 1:** Comparison of hospitals of the same size and ERAS Implementation.

Characteristics	ERAS-A (*n* = 2 hospitals)	*versus*	Non-ERAS-A (*n* = 3 hospitals)	*P*	ERAS-NA† (*n* = 4 hospitals)	*versus*	Non-ERAS-NA† (*n* = 4 hospitals)	*P*
Stays (*n*)	7523		11 892		15 707		16 871	
LoS (mean, days)	5.7		6.5	<0.001	5.5		6.2	<0.001
Low LoS outlier (%)	11.6		9.8	<0.001	6.6		5.8	<0.001
1-day DRG (%)	11.5		7.5	<0.001	11.1		8.9	<0.001
Costs (mean, Euros)	15 322		19 176	<0.001	12 298		13 518	<0.001
Revenue (mean, Euros)	12 740		14 414	<0.001	10 836		12 229	<0.001
Coverage rate (%)	86		79	<0.001	88.2		90.6	<0.001

†Non-academic hospitals totaling more than 2500 discharges per year. ERAS-A, enhanced recovery after surgery (ERAS) certified academic (A) hospitals deploying ERAS in their surgical department; ERAS-NA, enhanced recovery after surgery (ERAS) certified non-academic (NA) hospitals deploying ERAS in their surgical department; LoS, length of hospital stay; DRG, diagnosis-related groups.

The present results corroborate the findings of several large-scale studies showing that ERAS programmes decrease the postoperative LoS of surgical patients^4,5^, and confirmed ERAS as an efficient perioperative management pathway, as these protocols reduce variability in care, streamline processes, and improve outcomes. The mean cost per stay in ERAS-A hospitals was over 3800 Euros lower compared to non-ERAS-A hospitals and 1220 Euros in non-academic hospitals. However, due to financial issues specifically related to SwissDRG, the short LoS in ERAS hospitals resulted in a reduced reimbursement amount, leading to a loss of revenue for ERAS hospitals. Although costs are significantly lower in ERAS hospitals, the mean margin per case remains negative, in part due to the adjusted (reduced) reimbursement amount.

From a healthcare system point of view, the lower costs and reimbursement amounts induced by ERAS hospitals represent substantial savings. However, as less than 14% of hospitals deployed ERAS in Switzerland, the real cost-saving potential revealed by the present study remains unexploited. Despite differing reimbursement systems, comparable costs make the issue of missed saving opportunities of non-ERAS hospitals warrant further studies across healthcare settings.

The present study highlights important disincentives for wider ERAS deployment and sustainability in Switzerland, despite undeniable clinical benefits and cost-saving opportunities. It is therefore mandatory to adapt payment mechanisms and compensate for the loss of revenue induced by high-performing ERAS hospitals. Ultimately, the healthcare system could unlock substantial savings while improving the quality of care.

## Supplementary Material

zraf017_Supplementary_Data
