# Supplementary material for: Financial incentives for the deployment of Enhanced Recovery After Surgery (ERAS) in the SwissDRG inpatient prospective payment system: a national study
Source: BJS Open. 2025 Mar 3;9(1):zraf017. doi: 10.1093/bjsopen/zraf017 (PMC11879281; doi:10.1093/bjsopen/zraf017)
Supplement: zraf017_Supplementary_Data [file zraf017_supplementary_data.docx]

**Financial incentives for the deployment of Enhanced Recovery After Surgery (ERAS) in the SwissDRG inpatient prospective payment system: A national study**

Gaëtan-Romain Joliat^1*^, Fabian Grass^1*^, Joachim Marti^2^, Valérie Addor^3^, Lucien Gardiol^4^, Charles André Vogel^4^, Nicolas Demartines^5^, Fabio Agri^1,4^.

*Co-first authors.

^1^ Department of Visceral Surgery, Lausanne University Hospital CHUV, University of Lausanne (UNIL), Lausanne, Switzerland.

^2^ Center for Primary Care and Public Health (Unisanté), University of Lausanne, Lausanne, Switzerland

^3^ Department of Development and External Affairs, Lausanne University Hospital CHUV, Lausanne, Switzerland.

^4^ Department of Administration and Finance, Lausanne University Hospital CHUV, Lausanne, Switzerland.

^5^ General direction, Lausanne University Hospital CHUV, Lausanne, Switzerland.

**Corresponding author**

Gaëtan-Romain Joliat, MD, PhD

Department of Visceral Surgery

Lausanne University Hospital CHUV

Rue du Bugnon 46

1011 Lausanne, Switzerland

Phone: +41 79 556 42 93

Fax: +41 21 314 23 11

Email: [gaetan-romain.joliat@chuv.ch](mailto:gaetan-romain.joliat@chuv.ch)

**Supplementary Materials - Index**

| **Supplementary Methods** |  |
| --- | --- |
| ERAS vs non-ERAS hospitals  SwissDRG | *page 2*  *page 2* |
| National benchmarking registry  Readmission within 18 days post-discharge | *page 2*  *page 2* |
| **Supplementary Results** |  |
| Overall comparison | *page 4* |
| Comparison of hospitals of same size and scope | *page 4* |
| **References** | *page 5* |
|  |  |

**Supplementary Methods**

*ERAS vs non-ERAS hospital*

ERAS hospitals were defined as ERAS certified hospitals deploying the continuously audited enhanced recovery program in their surgical department [1]. First, for descriptive purposes, an overall comparison between ERAS and non-ERAS hospitals was realized. Then, to consider the scope and size of each hospital, similar institutions were compared based on their size and scope. Therefore, academic hospitals deploying ERAS (ERAS-A) were compared to academic hospitals not deploying ERAS (non-ERAS-A), and non-academic hospitals deploying ERAS (ERAS-NA) were compared to same size and scope non-academic hospitals not deploying ERAS (non-ERAS-NA). Non-academic hospitals include public regional and city hospitals as well as private clinics.

*SwissDRG*

Swiss Diagnosis-Related Groups (DRGs) are obtained by combining diagnostic codes (10th edition of the German modified international classification of disease), procedures codes (CHOP codes based on the Swiss classification of interventions) and demographic holdout data such as age, gender and type of admission.

Due to the extensive ERAS experience in the field of digestive surgery, only Swiss DRGs of major diagnostic categories (MDC) 06 and 07 corresponding to diseases and disorders of the digestive organs and of the hepatobiliary system and the pancreas, respectively, were taken into consideration for this study [2]. In 2022, 1’279’692 SwissDRG codes were reported, of which 159’365 (12.5%) from MDC 06 and 07 [3].

Depending on the LoS, a DRG can either be an inlier, ensuring to the hospital the fixed fee set for that DRG, or an outlier triggering a fee adjustment. A stay with a statistically defined “normal” LoS for a given DRG is therefore an inlier. However, if the LoS is lower than the limit set for that given DRG, the case is considered a low LoS outlier, triggering a per diem financial deduction taken from the fee set for inliers in that DRG. However, not all DRGs have a lower limit. Moreover, a 1-day DRG is a DRG that captures previous years’ frequent low LoS outliers and converts them into inliers. According to official Swiss national calculation method, LoS corresponds to the number of nights spent during the stay. Only stays lasting 1 day (spending 1 night) are included in a 1-day DRG.

Revenues (in CHF) were calculated by multiplying DRG’s CW by a BR. To simplify, the BR, yearly negotiated by each hospital and therefore slightly varying among them, was set at CHF 10’650 for academic hospitals, CHF 10’000 for non-academic hospitals and CHF 10’400 for the overall comparison. Mean margin per case was defined as mean costs per case minus mean revenue per case. Coverage rates were defined as mean costs per case divided by the mean revenue per case multiplied by 100 and were presented as percentages.

DRGs, CW, costs, revenues, LoS and low LoS outliers were available from the SpitalBenchmark database.

*National benchmarking registry*

Data were collected from a national platform (SpitalBenchmark) aiming to promote healthcare transparency and comparison of inpatient costs in Switzerland [4]. Swiss hospitals and clinics voluntarily participating in this benchmarking registry are members of the SpitalBenchmark association [4]. In 2022, out of 278 hospitals in Switzerland, 138 (49.6%) provided acute somatic care [5,6]. Overall, 248/278 (89.2%) hospitals were members of the association in 2022 and contributed to this registry. The proportion of members and non-members was 132/138 (95.6%) among the acute somatic hospitals [5,6]. Inpatient stays falling in a DRG of interest were therefore reviewed and compared among these 132 acute somatic hospitals contributing to the national registry.

*Readmission within 18 days post-discharge*

The readmission rate is not routinely collected by the Spitalbenchmark database, and therefore is not explicitly reported. Nonetheless, the study considers readmissions within 18 days, which are consolidated into a single DRG under a Swiss billing rule if the readmission falls under the same Major Diagnostic Category (MDC) as the initial stay (i.e., the readmission is linked to and considered part of the original stay). As a result, readmission costs are included in the overall stay and not accounted for separately.

**Supplementary Results**

*Overall comparison*

A total of 15/111 (13.5%) hospitals were ERAS hospitals. They accounted for 40’364 inpatient stays overall, while non-ERAS hospitals accounted for 102’391 inpatient stays in 2022. Among ERAS hospitals (N=15), there were two (13%) ERAS-A hospitals and 13 (87%) ERAS-NA hospitals, including regional hospitals (N=10), city hospitals (N=2) and one clinic. Non-ERAS hospitals (N=96) also included 3 non-ERAS-A hospitals (3%) and 93 (97%) non-ERAS-NA hospitals, including regional hospitals (N=29), city hospitals (N=27) and clinics (N=37). Mean coverage rates were 93.5% for ERAS and 94.6% for non-ERAS (p<0.001).

*Comparison of hospitals of same size and scope*

*Academic hospitals*

In the online database, 5 academic hospitals were included totaling 19’415 hospital stays. Two were ERAS-A hospitals (7’523, 39%) and 3 non-ERAS-A hospitals (11’892, 61%). Mean costs per case were lower among ERAS-A hospitals (CHF 14’402) than among non-ERAS-A hospitals (CHF 18’024, p<0.001). Similar results were found for mean revenues per case (ERAS-A: CHF 11’989 vs. non-ERAS-A: CHF 13’564, p<0.001). ERAS-A patients had a shorter mean LoS (5.7 days) compared to non-ERAS-A patients (6.5 days, p<0.001). Rates of inliers were similar in ERAS-A and non-ERAS-A hospitals (6’080/7’523=80.8% vs. 9’508/11’892=80.0%, p=0.140). The rates of low LoS outliers and of 1-day DRG were both higher in ERAS-A hospitals (876/7’523=11.6% and 864/7523=11.5% in ERAS-A hospitals vs. 1’164/11’892=9.8% and 896/11’892=7.5% in non-ERAS-A hospitals, p<0.001).

Coverage rates were 86% for ERAS-A hospitals and 79% for non-ERAS-A hospitals (p<0.001).

*Sample of non-academic hospitals*

Regional hospitals totaling more than 2’500 discharges per year and grouped in a DRG of the MDC 06 or 07 were compared. Four ERAS-NA hospitals accounting for 15’707 stays were compared to 4 non-ERAS-NA hospitals accounting for 16’871 stays. Mean costs per case were lower among ERAS-NA hospitals (CHF 11’559) compared to non-ERAS-NA hospitals (CHF 12’706, p<0.001). Similar results were found for mean revenue per case (ERAS-NA: CHF 10’197 vs. non-ERAS-NA: CHF 11’508, p<0.001).

The rates of low LoS outliers and of 1-day DRGs were both higher in ERAS-NA hospitals (1’040/15’707=6.6% and 1’742/15’707=11.1% in ERAS-NA hospitals vs. 973/16’871=5.8% and 1’501/16’871=8.9% in non-ERAS-NA hospitals (p<0.001).

Coverage rates were 88.2% for ERAS regional hospitals and 90.6% for non-ERAS regional hospitals (p<0.001).

**References**

1. ERAS Society website [Internet]. [cited 2024 Apr 28]. Available from: <https://erassociety.org/>.

2. Swiss DRG MDC categories 06 and 07 [internet]. [cited 2024 Apr 28]. Available from: <https://www.swissdrg.org/application/files/1316/3878/5723/SwissDRG-Version_11.0_Fallpauschalenkatalog_AV_2022_2022_f.pdf>

3. FSO (Federal Statistical Office). Hospital medical statistics: number of cases by age groups, according to the SwissDRG patient group classification. FSO website [internet]. [cited 2024 July 7]. Available from: <https://www.bfs.admin.ch/bfs/fr/home.assetdetail.28625244.html>

4. SpitalBenchmark [internet]. [cited 2024 July 7]. Available from: <https://www.spitalbenchmark.ch/>

5. SpitalBenchmark. Cost and performance comparison between hospitals. Benchmarking procedure for Swiss hospitals 2023 - Based on costs for the 2022 financial year [internet]. [cited 2024 July 7]. Available from: [Benchmarkingverfahren_Schweizer_Spitäler_2023_def_1.0.pdf (spitalbenchmark.ch)](https://www.spitalbenchmark.ch/fileadmin/user_upload/Benchmarkingverfahren_Schweizer_Spit%C3%A4ler_2023_def_1.0.pdf)

6. FOPH (Federal Office of Public Health). Key Figures of Swiss Hospitals 2022 - Chiffres clés des hôpitaux suisses 2022 [internet]. [cited 2024 July 7]. Available from: [kzp22_publication.pdf (bagapps.ch)](https://spitalstatistik.bagapps.ch/data/download/kzp22_publication.pdf?v=1710771312)
